# Prevalence of Human Intestinal *Entamoeba* spp. in the Americas: A Systematic Review and Meta-Analysis, 1990–2022

**DOI:** 10.3390/pathogens11111365

**Published:** 2022-11-16

**Authors:** Andrea Servián, Elisa Helman, María del Rosario Iglesias, Jesús Alonso Panti-May, María Lorena Zonta, Graciela Teresa Navone

**Affiliations:** 1 Centro de Estudios Parasitológicos y de Vectores (CEPAVE-CONICET-UNLP), Boulevard 120, La Plata 1900, Buenos Aires, Argentina; 2 Facultad de Ciencias Veterinarias, Universidad Nacional de La Plata, 60 y 118, La Plata 1900, Buenos Aires, Argentina; 3 Laboratorio de Inmunoparasitología (LAINPA), Facultad de Ciencias Veterinarias, Universidad Nacional de La Plata (FCV-UNLP), La Plata 1900, Buenos Aires, Argentina; 4 Centro de Investigaciones Regionales “Dr. Hideyo Noguchi”, Universidad Autónoma de Yucatán, Avenida Itzáes, No. 490 x Calle 59, Centro, Merida C.P. 97000, Yucatan, Mexico

**Keywords:** *Entamoeba*, the Americas, human infection

## Abstract

Among the seven species of *Entamoeba* known to infect humans, *E. histolytica* is widely recognized as a pathogen. It is reported that *Entamoeba* infections are common in the developing world, but rare in developed countries. The best way to diagnose these protozoan parasites is to detect antigens or DNA in the stool. This study aimed to review the prevalence, distribution, and diagnosis methods of *Entamoeba* spp. infecting humans in the Americas between 1990 and 2022. A systematic review and meta-analysis were performed, including 227 studies on *Entamoeba* infections from 30 out of 35 American countries. The pooled prevalence of each species of *Entamoeba* was calculated using the random-effects model. The assignment of *Entamoeba* species was mainly performed by microscopy. The most widely distributed and prevalent species was *E. coli* (21.0%). Of the studies, 49% could not differentiate the species of the *Entamoeba* complex. The pathogenic species *E. histolytica* was distributed among 22 out of 30 American countries studied, with a pooled prevalence of 9%. Molecular data on *Entamoeba* species are still scarce. This is the first study that reviewed and summarized data on the prevalence of this protozoan genera among American countries.

## 1. Introduction

The genus *Entamoeba* includes unicellular, anaerobic, and parasitic organisms, which infect humans, nonhuman primates, and other vertebrate and invertebrate species worldwide [1]. To date, this genus includes at least seven species that infect the human intestinal lumen: *E. histolytica*, *E. dispar*, *E. moshkovskii*, *E. bangladeshi*, *E. coli*, *E. hartmanni,* and *E. polecki*. The first four species have morphologically identical cysts and trophozoites. Although only *E. histolytica* has been well recognized as a causative agent of intestinal and extraintestinal amoebiasis [2], *E. moshkovskii* has been described as a potential pathogen by the latest studies [3,4]. Moreover, some strains of *E. dispar* were able to produce liver and intestinal lesions and *E. bangladeshi* has been discovered in cyst-containing diarrheal samples [5,6]. On the other hand, infections with *E. polecki* and *E. hartmanni,* whose cysts may be confused with immature states of *E. histolytica,* are rare and/or not associated with any disease [7]. In addition to these species known to infect people, *E. nuttalli*, which is prevalent in nonhuman primates, was detected in a caretaker at a zoo [8].

*Entamoeba histolytica* is the pathogenic species responsible for amoebiasis throughout the world [9]. The Global Burden of Disease 2010 Study estimates that amoebiasis accounts for 2.2 million disability-adjusted life years and around 55,500 annual deaths [10,11]. In addition, amoebiasis is common in developing countries and affects predominantly individuals with poor socioeconomic conditions, unhygienic practices, and/or malnutrition [12]. 

*Entamoeba* parasites are cosmopolitan, except for *E. moshkovskii*, which is endemic in Bangladesh, North America, and South Africa, and *E. bangladeshi*, which has also been found in the last two regions mentioned [5,13,14]. In general, *Entamoeba* infection is mostly seen in people living or traveling to tropical and subtropical areas (Asia, Africa, India, Indonesia, Mexico, South America, or South Africa) [13,15,16]. Taking into account that some species are indistinguishable by microscopy, a specific and accurate method of diagnosis is required. The identification, diagnosis, and characterization of *Entamoeba* have been based mainly on the microscopy method [17], which cannot differentiate true infections caused by *E. histolytica* from nonpathogenic *Entamoeba* spp. [11]. Among the features for the differentiation of *Entamoeba* species, the main ones are the cyst size, the number of nuclei, and the appearance of the peripheral chromatin; however, some of these may not always be discernible by light microscopy of fecal concentrates. In addition, the existence of mature as well as immature cysts and morphologically identical *Entamoeba* species can confuse the diagnostic criteria [7,18]. In fact, light microscopy is considered less reliable to identify the species of *Entamoeba* than either culture, antigen detection tests, and antibody-based stool ELISA [19,20].

Lately, molecular tools, including conventional PCR, nested PCR, real-time PCR, multiplex PCR, and loop-mediated isothermal amplification assay (LAMP) [21], have been developed and are increasingly used for the detection and differentiation of *Entamoeba* species in fecal samples. Since 1990, these methods have been implemented and contributed to reevaluating the taxonomy, epidemiology, and clinical significance of *Entamoeba* isolates found in human fecal samples [20,22]. However, molecular techniques require specific equipment and trained technical staff. In resource-poor regions, the high cost of these methods precludes their use. Thus, PCR- based methods have only been performed in reference and research laboratories of wealthy countries and are not available to the most exposed population [23].

Despite many American countries, mainly those characterized by poor sanitation and socioeconomic conditions, having been reported as endemic for amoebiasis [12], the prevalence of infection, discriminated by species of *Entamoeba,* is scarcely known. Particularly, amoebiasis is one of the 20 main causes of disease in Mexico; however, some isolated epidemiological studies have been made using molecular tools to characterize *E. histolytica* and *E. dispar* [24,25,26,27]. In South America, several studies performed microscopic diagnosis of *Entamoeba* species, but discriminatory studies between species are relatively scarce. In the United States, California and Texas have shown a higher rate of amebiasis-related mortality [28]. Although there are data on the frequency of human *Entamoeba* infections in the Americas, there is no global analysis of the prevalence and distribution of this protozoan by geographic area, age group, and method performed for its diagnosis. Therefore, this investigation aimed to review the prevalence, distribution, and diagnosis methods of *Entamoeba* spp. Infecting humans in the Americas between 1990 and 2022.

## 2. Material and Methods

### 2.1. The Review Question

What are the prevalence, geographical distribution, and diagnosis methods of intestinal *Entamoeba* species in humans from different American countries?

### 2.2. Literature Research

A literature review of published articles on human infections with *Entamoeba* species was conducted using electronic databases (BioOne, Google Scholar, JSTOR, PAHO IRIS, PubMed, Scopus and WHO IRIS). Search terms included but were not limited to “*Entamoeba*”, “amoebiasis”, “human infections”, “GenBank”, “prevalence”, and “America”. 

The search was conducted between November 2021 and July 2022. The review included studies on human infections with *Entamoeba* without restrictions on language. From each study, the following data were extracted: number of samples, prevalence, and technique performed for diagnosis, country, year, type of study, and references. 

Articles were included if they described human infections with intestinal *Entamoeba* species from American countries published from 1990 to 2022. However, they were excluded if they did not describe human infections, did not provide the geographic location, the number of infected subjects, and/or the diagnosis technique. Case reports, letters, editorials, subject reviews, meta-analyses, special theme papers, and symposium proceedings were excluded. This review only included descriptive epidemiological studies evaluating the prevalence of species of *Entamoeba* in humans. 

### 2.3. Data Summary 

The random-effects meta-analysis model was used to analyze the prevalence of the *Entamoeba* species. The heterogeneity among studies was evaluated using the package *meta* implemented in R software version 4.2.1 [29,30]. The I^2^ is expressed as a proportion of the total variance and ranges from 0 to 100%, with values of 25%, 50%, and 75% suggested to represent low, moderate, and high levels of heterogeneity, respectively [31]. Stratified meta-analyses were performed according to regions and techniques. 

Maps were performed using QGIS version 3.12 [32].

### 2.4. GenBank Sequences

We summarize the GenBank nucleotide sequences of intestinal *Entamoeba* isolates obtained, available on the website: https://www.ncbi.nlm.nih.gov/nuccore (accessed on 1 September 2022).

## 3. Results

Our systematic literature search retrieved 1935 manuscripts for further evaluation. The screening according to our selection criteria left 227 articles for detailed review (Supplementary Table S1). Brazil was the country better characterized by the high number (N = 60) of studies distributed throughout almost its entire territory. Other countries better represented were Argentina (N = 34), Mexico (N = 23), and Colombia (N = 20) (Supplementary Table S1). The least represented countries were Paraguay (N = 4), Costa Rica (N = 2), and Canada (N = 1), among others. No prevalence data were found for any species of *Entamoeba* in Uruguay, Suriname, the Dominican Republic, Bahamas, Barbados, and Trinidad and Tobago. 

The results of this search strategy are presented according to the Preferred Reporting Items for Systematic Reviews and Meta-Analyzes (PRISMA) flowchart (Supplementary Figure S1). Data were extracted according to the PRISMA Statement [33].

**Figure 1:**
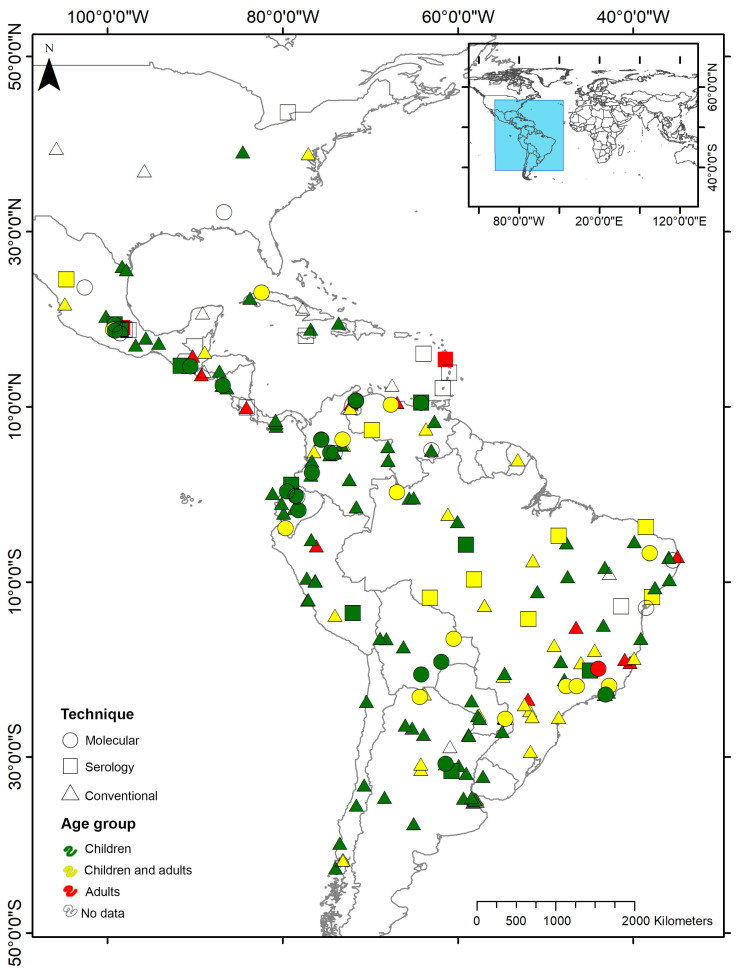
Maps representing the distribution of the studies included, according to the species detected, the techniques performed and age groups. The techniques performed are represented by different figures, and the age groups are represented by different colors.

Of the 227 studies included, 70.5% (160/227) identified intestinal amoeba by conventional microscopic diagnosis, 8.8% (20/227) by molecular characterization, 4.8% (11/227) by serology, and 0.4% (1/227) by zymodeme analysis. Some studies performed multiple techniques to confirm the diagnosis: 6.2% (14/227) by microscopic and serology, 8.8% (20/227) by microscopy and molecular diagnosis, 0.4% (1/227) by molecular and serology tests, and 0.9% (2/227) by serology, molecular, and microscopic diagnosis. Of the total of studies that performed conventional microscopic-based techniques (N = 196), 73.0% employed sedimentation methods (formalin-ether concentration technique, spontaneous sedimentation, Ritchie modified, Faust method, and flotation techniques), 7.1% sedimentation and trichrome staining, and 14.8% performed direct smear observations. These conventional techniques were carried out in most American countries, while molecular diagnoses were restricted to some of them, mainly in Brazil, Colombia, and Ecuador (Figure 1).

Of the 227 studies analyzed, 198 (86.8%) distributed samples by age group. Among them, 59.1% (117/198) were performed in children, 29.8% (59/198) in both children and adults, and 11.1% (22/198) exclusively in adults. Concerning the latter, these studies were performed on specific populations, such as immunocompromised individuals (chronic renal and HIV patients), pregnant women, migrants, food handlers, and blood donors, among others. In addition, these studies were mainly carried out in Brazil, and there were some records in Venezuela, Peru, and Central American countries (Figure 1). 

Forty-nine percent of the studies (112/227) were unable to differentiate the species *E. histolytica*, *E. dispar*, and *E. moshkovskii* and determined the diagnosis as *Entamoeba* complex. On the other hand, a small number of studies (3.1%; 7/227) were unable to determine any species and defined the diagnosis as *Entamoeba* spp. 

**Figure 2:**
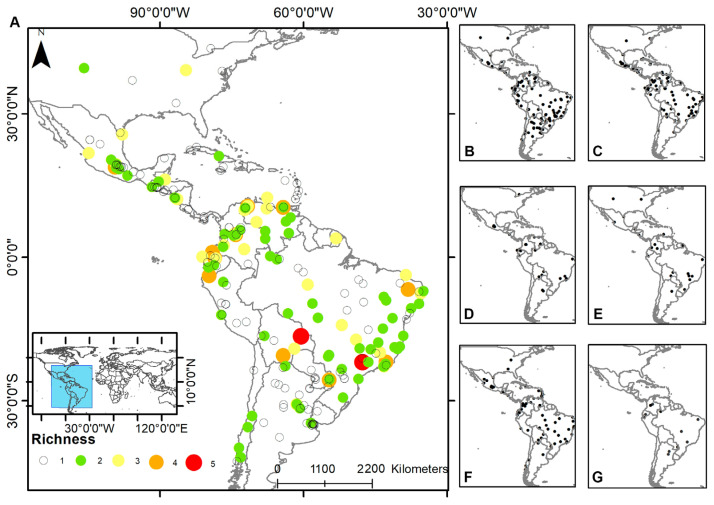
Map showing (**A**) the richness (total of *Entamoeba* species detected in each study) represented by different colors and sizes of circles. On the right, each map represented the distribution of (**B**) *E. coli*, (**C**) *E. complex*, (**D**) *E. dispar*, (**E**) *E. hartmanni*, (**F**) *E. histolytica*, (**G**) *Entamoeba* spp., *E. polecki* and *E. moshkovskii*.

Half of the studies (114/227) found only one species of the *Entamoeba* genus, and two (0.9%; 2/227) described five different ones (*E. histolytica*, *E. dispar*, *E. coli*, *E. hartmanni*, *Entamoeba* complex/ *Entamoeba* spp.) (Figure 2). Around sixty percent of the studies (61.7%; 140/227) detected *E. coli*, 49.3% (112/227) *E. complex* and 36.1% (82/227) *E. histolytica*. Few studies found *E. dispar* (13.2%; 30/227), *E. polecki* (0.9%; 2/227), and *E. moshkovskii* (1.3%; 3/227). *Entamoeba coli* was recorded in all American countries where studies were carried out (except in Canada). A similar geographical distribution was determined for the *Entamoeba* complex and *E. histolytica*, with few records in Argentina. In contrast, *E. dispar* was recorded in a lower number of countries, *E. polecki* was only detected in Ecuador and Argentina, and *Entamoeba moshkovskii* was diagnosed in Colombia and Venezuela. The distribution of *E. hartmanni* was limited to a few records in Venezuela, Colombia, Ecuador, Bolivia, Brazil, Argentina, French Guiana, Mexico, Nicaragua, Belize, and the United States (Figure 2).

**Figure 3:**
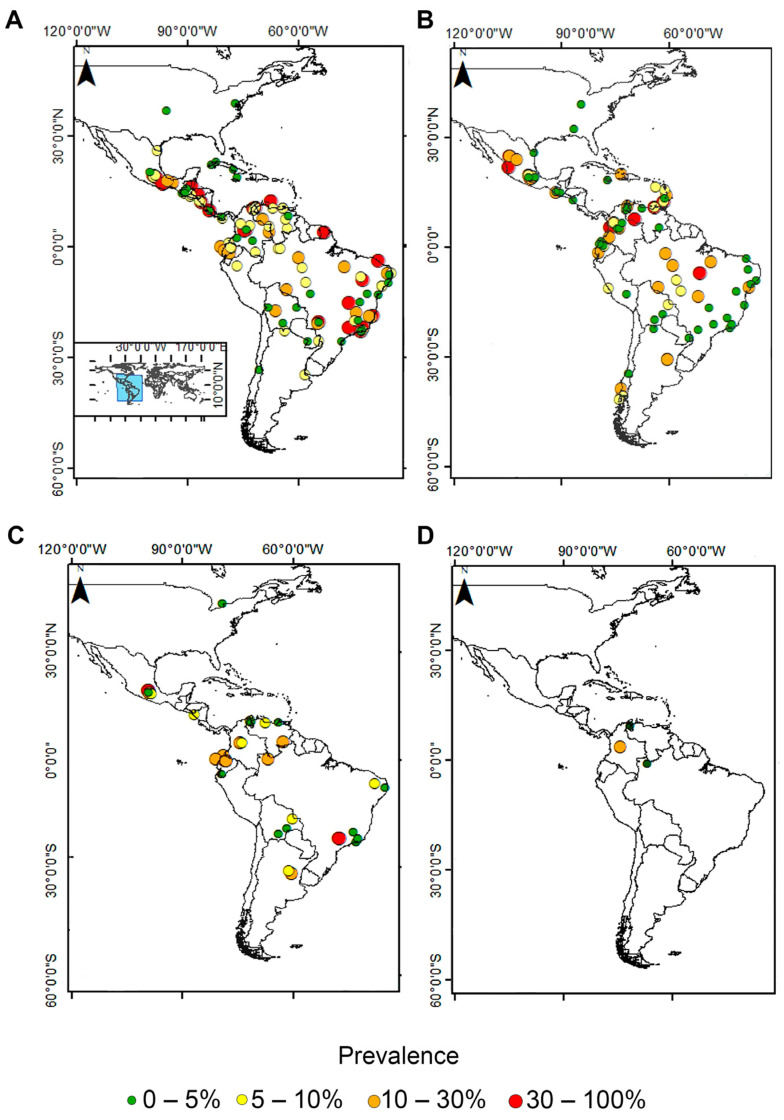
Maps showing the prevalence of *Entamoeba* species, which are morphologically identical, recorded among American countries studied: (**A**) *Entamoeba* complex, (**B**) *E. histolytica*, (**C**) *E. dispar*, and (**D**) *E. moshkovskii*.

Regarding the prevalence estimated for each species, different ranged values were reported among American countries. Globally, the infection with the *Entamoeba* complex in the analyzed studies ranged from 0.29% (2/595) to 100% (106/106). Particularly, the *Entamoeba* complex was reported in high prevalence in Mexico, Ecuador, Venezuela, Colombia, Bolivia, and Brazil (Figure 3A). The prevalence of *E. histolytica* in the analyzed studies ranged from 0.08% (5/6289) to 82.6% (57/69), being more frequent in Venezuela, Colombia, and Mexico (Figure 3B). On the other hand, the prevalence of *E. dispar* in the analyzed studies was between 0.44% (4/903) and 88% (106/120) by specific methods (molecular and serology), and the only study performed by microscopy determined a prevalence of 14.6% (16/110) (Supplementary Table S1). In particular, high prevalence values of *E. dispar* were determined in Ecuador and Mexico (Figure 3C and Supplementary Table S1). The prevalence of *E. moshkovskii* was detected by molecular analysis and ranged from 1 to 25.4% (Figure 3D). The prevalence of *E. coli* reported in the analyzed studies was between 1.1% and 78%.

**Figure 4:**
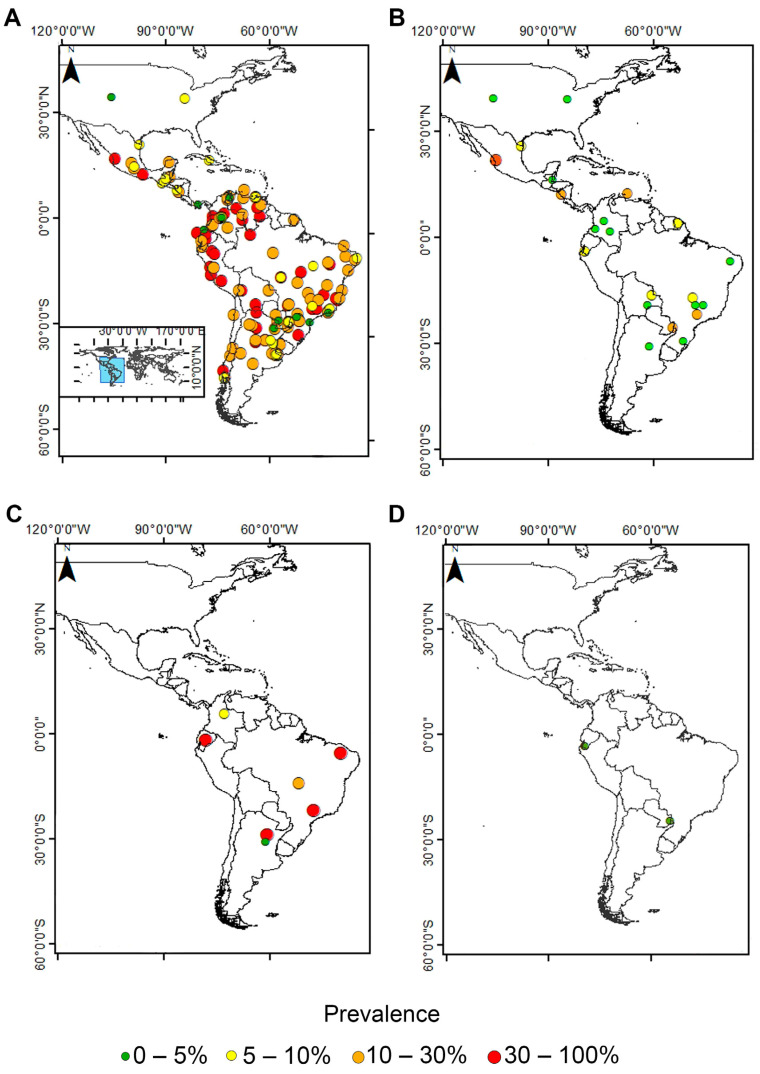
Maps showing the prevalence of each *Entamoeba* species registered among American countries studied: (**A**) *E. coli*, (**B**) *E. hartmanni*, (**C**) *Entamoeba* spp., and (**D**) *E. polecki*.

The prevalence of the nonpathogenic *E. coli* ranged from 1.1 (29/2604) to 78.0% (71/91) (Figure 4A). *Entamoeba hartmanni* prevalence ranged from 0.04% (1/2694) to 45.5% (139/306) (Figure 4B). The determination as *Entamoeba* spp. ranged from 3.04% (9/296) to 57.9% (89/154) (Figure 4C). Meanwhile, *E. polecki* was detected in two studies with very low frequency (0.3 and 0.5%) (Figure 4D).

### 3.1. Pooled Prevalence of Entamoeba Infection

Included studies were highly and significantly heterogeneous according to I^2^ statistics (>75%; *p* < 0.05), and therefore, random-effects models were used for the meta-analysis to synthesize pooled estimates of the prevalence of *Entamoeba* species.

Due to the variability in the number of studies for each country, stratified meta-analyses were performed on data divided into three subgroups according to geographical regions: (i) North and Central American countries (Nicaragua, Mexico, United States, Guatemala, Belize, Costa Rica, Cuba), (ii) Brazil, and (iii) the other South American countries (Argentina, Bolivia, Chile, Colombia, Ecuador, French Guiana, Paraguay, Peru, and Venezuela). 

**Figure 5:**
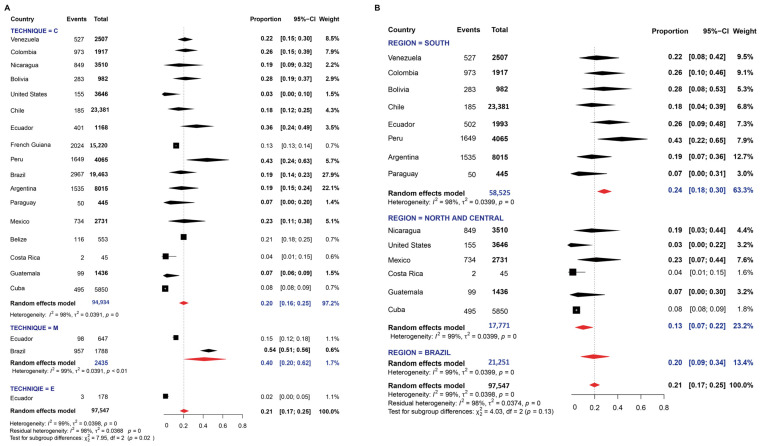
Forest plot for a random-effect meta-analysis of the pooled prevalence of *E. coli* in the American population by: (**A**) techniques: C: conventional methods, based on microscopy; M: molecular method, based on DNA amplification; E: Elisa method, serology-based; (**B**) Region: North and Central American countries as ‘Group 1’ (Nicaragua, Mexico, United States, Guatemala, Belize, Costa Rica, Cuba), Brazil as ‘Group 2’, and the other South American countries as group 3 (Argentina, Bolivia, Chile, Colombia, Ecuador, French Guiana, Paraguay, Peru, and Venezuela). Diamonds constitute a representation that summarizes the studies of each country. The complete version is available in Supplementary Figure S2.

A total of 140 studies were evaluated for determining the prevalence of *E. coli.* Random-effects meta-analysis showed a pooled prevalence of 21.0% with an I^2^ value (99%) indicating high heterogeneity (Figure 5 and Supplementary Figure S2). The pooled prevalence detected by molecular analysis (40.0%) was significantly higher than conventional and serology ones (χ^2^ = 7.95; *p* = 0.02) (Figure 5A). Regarding regions, the pooled prevalence was similar between them, and no statistical differences were found (*p* > 0.05) (Figure 5B).

**Figure 6:**
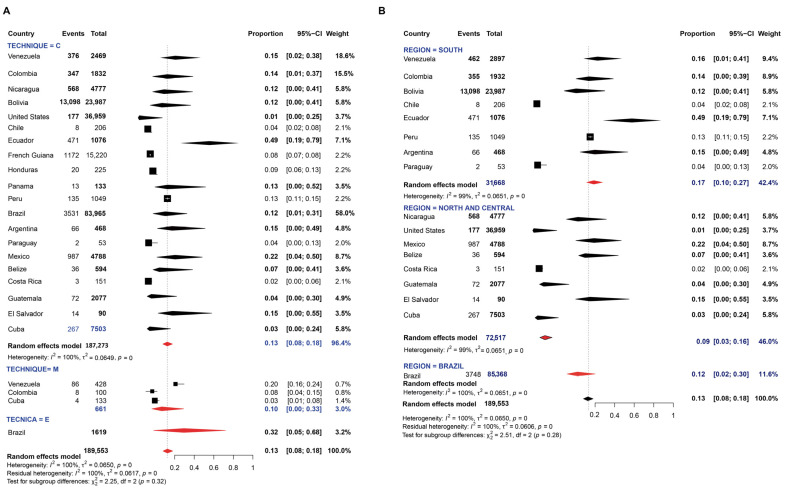
Forest plot for a random-effect meta-analysis of the pooled prevalence of the *Entamoeba* complex in the American population by: (**A**) techniques: C: conventional methods, based on microscopy; M: molecular method, based on DNA amplification; E: Elisa method, serology-based; (**B**) Region: North and Central American countries as ‘Group 1’ (Nicaragua, Mexico, United States, Guatemala, Belize, Costa Rica, Cuba), Brazil as ‘Group 2’, and the other South American countries as group 3 (Argentina, Bolivia, Chile, Colombia, Ecuador, French Guiana, Paraguay, Peru, and Venezuela). Diamonds constitute a representation that summarizes the studies of each country. The complete version is available on the Supplementary Figure S3.

A total of 110 studies were evaluated for determining the frequency of infection of *Entamoeba complex*. The random-effects pooled prevalence was 13.0% and the I^2^ was about 95% (Figure 6 and Supplementary Figure S3). The prevalence of this parasite was mainly determined by conventional methods and the meta-analyses revealed values ranging from 1 to 100% with an overall prevalence of 13%; the I^2^ value was 96.7%, indicating extremely high heterogeneity. Similar values of pooled prevalence were determined by DNA or serology based-detection methods, without statistical differences (Figure 6A). The pooled prevalence was higher in the group of South American countries (Figure 6B).

**Figure 7:**
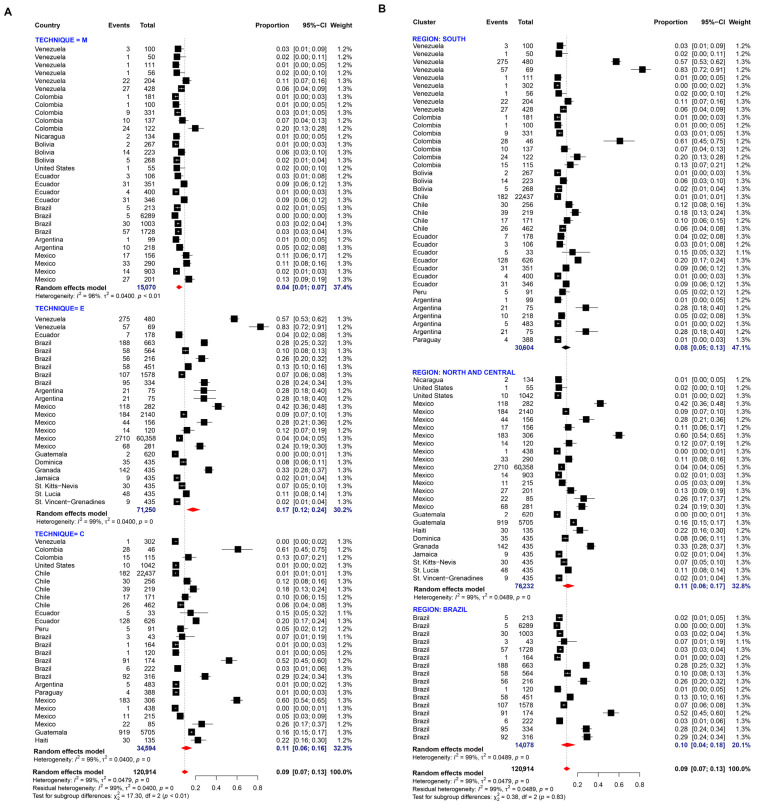
Forest plot for a random-effect meta-analysis of the pooled prevalence of *E. histolytica* in the American population by: (**A**) techniques: C: conventional methods, based on microscopy; M: molecular method, based on DNA amplification; E: Elisa method, serology-based; (**B**) Region: North and Central American countries as ‘Group 1’ (Nicaragua, Mexico, United States, Guatemala, Belize, Costa Rica, Cuba), Brazil as ‘Group 2’, and the other South American countries as group 3 (Argentina, Bolivia, Chile, Colombia, Ecuador, French Guiana, Paraguay, Peru, and Venezuela).

A sum of 80 studies were evaluated for determining the prevalence of *E. histolytica*. The random-effects pooled prevalence was 9.0% with a considerably high value of I^2^ (99%) (Figure 7). A higher pooled prevalence was determined by ELISA serology methods, followed by conventional ones (χ^2^ = 17.30, *p* < 0.01) (Figure 7A). The pooled prevalence of *E. histolytica* was higher in Brazil, without statistical differences among regions (*p* > 0.05) (Figure 7B).

**Figure 8:**
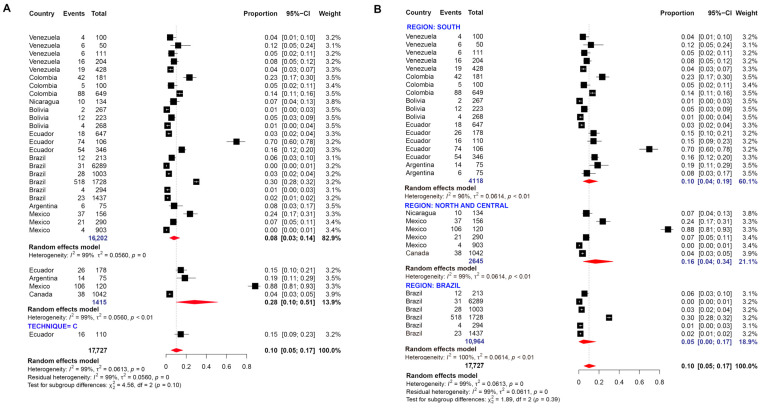
Forest plot for a random-effect meta-analysis of the pooled prevalence of *E. dispar* in the American population by: (**A**) techniques: C: conventional methods, based on microscopy; M: molecular method, based on DNA amplification; E: Elisa method, serology-based; (**B**) Region: North and Central American countries as ‘Group 1’ (Nicaragua, Mexico, United States, Guatemala, Belize, Costa Rica, Cuba), Brazil as ‘Group 2’, and the other South American countries as group 3 (Argentina, Bolivia, Chile, Colombia, Ecuador, French Guiana, Paraguay, Peru, and Venezuela).

Thirty studies were evaluated for determining the prevalence of *E. dispar.* Random-effects meta-analysis showed a pooled prevalence of 10.0% in American regions (Figure 8). The prevalence of this protozoan was mainly determined by molecular methods and the meta-analyses revealed values ranging from 1 to 70% with an overall prevalence of 8.0% (Figure 8A). However, this pooled prevalence was lower than that determined by microscopy or serology-based methods. Among regions, this prevalence was higher in North and Central American countries (Figure 8B).

**Figure 9:**
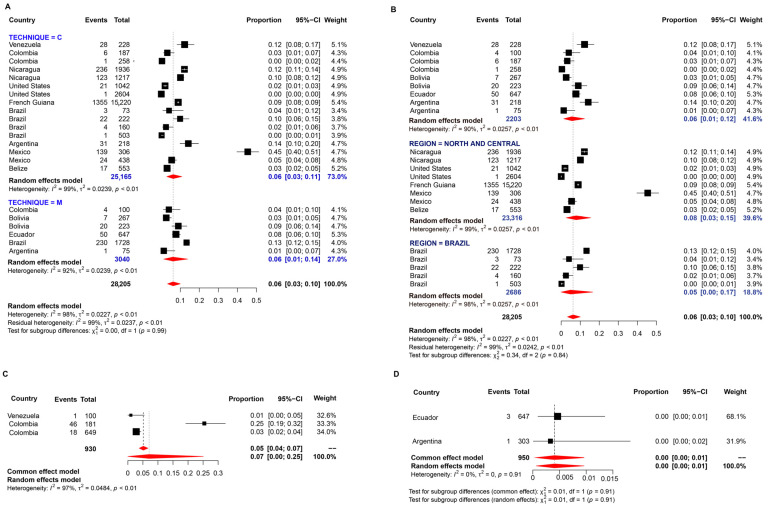
Forest plot for a random-effect meta-analysis of the pooled prevalence of *E. hartmanni* in the American population by: (**A**) techniques: C: conventional methods, based on microscopy; M: molecular method, based on DNA amplification; E: Elisa method, serology-based; (**B**) Region: North and Central American countries as ‘Group 1’ (Nicaragua, Mexico, United States, Guatemala, Belize, Costa Rica, Cuba), Brazil as ‘Group 2’, and the other South American countries as group 3 (Argentina, Bolivia, Chile, Colombia, Ecuador, French Guiana, Paraguay, Peru, and Venezuela); Forest plot for a random-effect meta-analysis of the pooled prevalence of (**C**) *E. moshkovskii* and (**D**) *E. polecki*.

Twenty-two studies were evaluated for determining the prevalence of *E. hartmanni.* The random-effects pooled prevalence was 6.0% and the I^2^ value was 98.0% indicating extremely high heterogeneity. The pooled prevalence was similar among techniques and regions, without statistical differences (Figure 9A,B).

Three studies were evaluated for determining the prevalence of *E. moshkovskii*. Random-effects meta-analysis showed a general pooled prevalence of 7.0% (Figure 9C).

Two studies were evaluated for determining the prevalence of *E. polecki*. Random-effects meta-analysis showed a general pooled prevalence of 0% (Figure 9D).

### 3.2. GenBank Sequences

The GenBank database contained scarce nucleotide sequence data about human *Entamoeba* species from American countries in the GenBank database. Only nucleotide sequences of SSU rRNA and tRNA genes were submitted (Table 1). Mexico and Brazil reported a higher number of sequences. 

## 4. Discussion

Globally, this review provides an up-to-date overview of the prevalence and distribution of *Entamoeba* species in 30 out of 35 American countries. Our results reflect a wide sampling of the different countries, but the southeastern areas, such as Brazil, are better represented since these regions present higher scientific production. High heterogeneity existed in the main meta-analysis for each *Entamoeba* species and persisted in the stratified analyses. 

According to our meta-analysis, *E. coli* was the most prevalent species (21.0%) and based on molecular methods the highest prevalence was obtained for this parasite (40%). However, more studies based on molecular tools are needed to corroborate if conventional methods overestimate the prevalence. In addition, its prevalence was higher in Brazil compared to the other American regions.

An accurate diagnosis of *E. histolytica* infection is important for patients with amoebic dysentery and asymptomatic infected individuals because it may easily be transmitted from person to person, especially in developing countries that have poor hygienic conditions and inadequate water treatment. In this review, the *Entamoeba* complex was the second most prevalent diagnosis recorded, being highly frequent in South American areas. Particularly, the higher prevalence rates of the *Entamoeba* complex were determined by conventional methods, while lower values were determined by serology or molecular analyses. This is probably a consequence of trophozoites of several other nonpathogenic intestinal amoebas or fecal macrophages, being misdiagnosed as *E. histolytica*/*E. dispar*/*E. moshkovskii* by morphological diagnosis [41,42]. Currently, molecular methods are recommended for distinguishing pathogenic *Entamoeba* species. However, most developing countries cannot afford to use PCR as a part of their diagnostic tool because it is technically complex and expensive, hence, microscopic examinations based on Wheatley trichrome staining have been the most commonly used method [43]. This systematic review showed that conventional methods have been the most widely used for the identification and assignment of *Entamoeba* organisms in American countries, but trichrome staining was only performed in 7.1% of the studies. 

It has been reported that *E. histolytica* and *E. dispar* infect around 10% of the world population [44]. However, Cui et al. [45] showed that the overall molecular prevalence of *Entamoeba* spp. was 3.5% in humans worldwide. They also showed that *E. histolityca* and *E. dispar* were responsible for 81.7% of this global prevalence, the latter being much more common than *E. histolytica* worldwide. Similarly, many studies reported that *E. dispar* infections were more frequent than *E. histolytica* [20,39,46,47]. This study showed that the diagnosis of *E. histolytica*, *E. dispar*, and *E. moshkovskii* was mainly based on molecular and serology methods. These techniques detected lower prevalence rates of these parasites compared to conventional methods. It is likely the frequency detected by microscopy overestimated the number of people infected with *E. histolytica*. Several studies have shown that the coproscopic diagnosis of this enteric protozoan is neither specific nor sensitive [46,47,48]. On the other hand, PCR based-assays avoid not only misdiagnosis but also overtreatment [49]. It is known that infections caused by *E. dispar* are much more common than *E. histolytica* worldwide [39]. Consistently, this review revealed that the pooled prevalence of *E. dispar* was higher than that of *E. histolytica*. However, a wider distribution of *E. histolytica* was determined compared to *E. dispar* and *E. moshkovskii*. The distribution of *E. moshkovskii* was limited to Venezuela and Colombia studies, which performed molecular methods. 

On the other hand, the distribution and number of studies that detected *E. hartmanni* were like *E. dispar*. The nonpathogenic species *E. hartmanni* can be distinguished from *E. histolytica*, *E. dispar*, and *E. moshkovskii* by optical microscopy. However, this distinction needs detailed observation of nuclear structures, which requires permanent smear staining, an ocular micrometer, and trained parasitologists. Therefore, the possibility of *E. hartmanni* infection should also be considered in people who excrete indistinguishable *E. histolytica/E. dispar/E. moshkovskii* complex and *E. hartmanni* cysts [17]. In this review, we observed that the identification of this nonpathogenic protozoan was mostly performed by microscopic diagnosis. 

There are several methods for the diagnosis of amoebiasis, each with different levels of sensitivity and specificity. Although many experts now consider microscopic diagnosis obsolete due to its low sensitivity, it is still employed in developing countries because of the lack of facilities to use other advanced methods [50]. Regarding ELISA tests, antigen-based methods have given good sensitivity and specificity, but serology includes limitations such as false negatives in early infections [51]. PCR is the most reliable diagnostic tool for the detection of *Entamoeba* species, and it is particularly useful for distinguishing pathogenic versus nonpathogenic ones [52]. Although these methods have been implemented since 1990, this review showed that their use is not yet frequent in the Americas. In addition, in the majority of studies, the *Entamoeba* assignment to species performed by microscopy and serology overestimated the rates of prevalence, which is in the cases of *E. histolytica* and *E. dispar*. 

Concerning *Entamoeba* species distribution through America, *E. coli* seemed to be the more cosmopolitan species. Its diagnosis by conventional methods is more easily performed than the diagnosis of the other species. Although this is a commensal parasite, it has the same transmission route as that of not only *Entamoeba* pathogenic species, but also protozoa such as *Giardia lamblia* and helminths. Thus, the frequency of *E. coli* should be used as an indicator of fecal/oral transmission, indicating intestinal parasite transmission through the water supply or contaminated food [53].

In contrast, the detection of species such as *E. polecki* and *E. moshkovskii* was limited to a few studies. *Entamoeba moshkovskii* was only detected in Venezuela and Colombia by using molecular methods. This species has long been thought of as a free amoeba, but in the last decade, it has been demonstrated that *E. moshkovskii* can infect humans and can be found more frequently in areas where amoebiasis shows high prevalence values [3]. Therefore, it is important to perform its diagnosis, especially when considering that it is morphologically indistinguishable from *E. histolytica*. On the other hand, *E. polecki* was detected in Ecuador by DNA detection and in Argentina by microscopy. 

Infections by protozoan parasites are typically associated with factors such as fecal contamination of food, limited access to safe drinking water, poor environmental sanitation, and vulnerable socioeconomic conditions [54]. In this sense, Latin America and the Caribbean remain the world’s most unequal regions, with 10% of the people still living in conditions of multidimensional poverty [55]. Given the socio-cultural features, these regions tend to have the highest rates of infection by parasites. Indeed, reports of infection with *Entamoeba* are higher in Latin America and the Caribbean than in North America. However, microscopy likely underestimates the frequency of infection, hence more studies which perform molecular methods are necessary to provide more accurate data on prevalence. 

## 5. Conclusions

This is the first study that reviewed and summarized data on the prevalence of *Entamoeba* species among American countries. 

*Entamoeba coli* was the most widely distributed species with high prevalence values in several American countries. Among species of the *Entamoeba* complex, *E. dispar* was the most prevalent. Moreover, it is important to point out that the prevalence of *E. histolytica* was high, indicating that this infection remains represent a significant health threat among American countries.

High heterogeneity was detected regarding the number of studies and techniques performed to diagnose *Entamoeba* species among American countries over around 30 years. This highlights the need to further investigate *Entamoeba* infections in the regions poorly represented. Moreover, since molecular methods are more reliable for *Entamoeba* diagnosis, more studies are needed to further expand our understanding of this parasite distribution and the diversity of these parasites in American regions. 

## Figures and Tables

**Table 1:** Nucleotide sequence data on *Entamoeba* species available in GenBank.

Molecular Marker	Species	Accession No.	Country	Host	Isolation Source	References
SSU rRNA gene	*Entamoeba dispar*	MK541026	Argentina	*Homo sapiens*	Stool	Direct submission
	*Entamoeba dispar*	OM985615	Argentina	*Homo sapiens*	Stool	Direct submission
	*Entamoeba dispar*	MZ787761	Argentina	*Homo sapiens*	Stool	Direct submission
	*Entamoeba dispar*	OM985618	Argentina	*Homo sapiens*	Stool	Direct submission
	*Entamoeba coli*	ON713469	Argentina	*Homo sapiens*	Stool	Direct submission
	*Entamoeba coli*	OM985619	Argentina	*Homo sapiens*	Stool	Direct submission
	*Entamoeba coli*	MZ787759	Argentina	*Homo sapiens*	Stool	Direct submission
	*Entamoeba coli*	OM985620	Argentina	*Homo sapiens*	Stool	Direct submission
	*Entamoeba coli*	MZ787760	Argentina	*Homo sapiens*	Stool	Direct submission
	*Entamoeba coli*	OM985617	Argentina	*Homo sapiens*	Stool	Direct submission
	*Entamoeba coli*	MK541024	Argentina	*Homo sapiens*	Stool	Direct submission
	*Entamoeba coli*	OM985616	Argentina	*Homo sapiens*	Stool	Direct submission
	*Entamoeba coli*	OM985619	Argentina	*Homo sapiens*	Stool	Direct submission
	*Entamoeba hartmanni*	MT703882	Argentina	*Homo sapiens*	Stool	[34]
	*Entamoeba polecki*	MH348163-MH348175	Argentina	*Sus scrofa domestica*	Stool	[35]
SSU rRNA gene	*Entamoeba dispar*	MW026767-MW026784	Brazil	*Homo sapiens*	Stool	[36]
	*Entamoeba histolytica*	MW026793 MW026794	Brazil	*Homo sapiens*	Stool	[36]
	*Entamoeba hartmanni*	MW026785-MW026792	Brazil	*Homo sapiens*	Stool	[36]
	*Entamoeba coli*	MW026735-MW026766	Brazil	*Homo sapiens*	Stool	[36]
	*Entamoeba coli*	FR686423	Brazil	*Homo sapiens*	Stool	[1]
tRNA	*Entamoeba dispar*	GU324326	Brazil	*Homo sapiens*	Stool	[37]
	*Entamoeba histolytica*	EF421375	Brazil	*Homo sapiens*	Stool	[38]
SSU rRNA gene	*Entamoeba histolytica*	KT825974	Colombia	*Homo sapiens*	Stool	[39]
	*Entamoeba moshkovskii*	KT825984-KT825993	Colombia	*Homo sapiens*	Stool	[39]
	*Entamoeba dispar*	KT825975-KT825983	Colombia	*Homo sapiens*	Stool	[39]
SSU rRNA gene	*Entamoeba coli*	FR686443	Peru	*Homo sapiens*	Stool	[1]
SSU rRNA gene	*Entamoeba coli*	FR686446	Ecuador	*Homo sapiens*	Stool	[1]
SSU rRNA gene	*Entamoeba hartmanni*	MK541027	Mexico	*Homo sapiens*	Stool	Direct submission
	*Entamoeba coli*	MK541025	Mexico	*Homo sapiens*	Stool	Direct submission
tRNA	*Entamoeba dispar*	GU324327-GU324329 GU324333- GU324337	Mexico	*Homo sapiens*	Mixed liver abscess	[37]
	*Entamoeba histolytica*	GU324330-GU324332	Mexico	*Homo sapiens*	Mixed liver abscess	[37]
	*Entamoeba histolytica*	JN191598 JN191599 JQ828978	Mexico	*Homo sapiens*	Cutaneous amoebiasis	[40]
	*Entamoeba histolytica*	KC791705-KC791758	Mexico	*Homo sapiens*	Amoebic liver abscess	[40]
	*Entamoeba dispar*	KX461938-KX461956	Mexico	*Homo sapiens*	Stool	[27]

## Data Availability

All data are incorporated into the article and its online Supplementary Materials.

## Supplementary Materials

The following supporting information can be downloaded at: https://www.mdpi.com/article/10.3390/pathogens11111365/s1, Figure S1: Flow diagram of the steps performed in this review; Figure S2: Forest plot for a random-effect meta-analysis of the pooled prevalence of E. coli in the American population by: (A) techniques: C: conventional methods, based on microscopy; M: molecular method, based on DNA amplification; E: Elisa method, serology-based; (B) Region: North and Central American countries as ‘Group 1’ (Nicaragua, Mexico, United States, Guatemala, Belize, Costa Rica, Cuba), Brazil as ‘Group 2’ and the other South American countries as group 3 (Argentina, Bolivia, Chile, Colombia, Ecuador, French Guiana, Paraguay, Peru and Venezuela); Figure S3: Forest plot for a random-effect meta-analysis of the pooled prevalence of the Entamoeba complex in the American population by: (A) techniques: C: conventional methods, based on microscopy; M: molecular method, based on DNA amplification; E: Elisa method, serology-based; (B) Region: North and Central American countries as ‘Group 1’ (Nicaragua, Mexico, United States, Guatemala, Belize, Costa Rica, Cuba), Brazil as ‘Group 2’ and the other South American countries as group 3 (Argentina, Bolivia, Chile, Colombia, Ecuador, French Guiana, Paraguay, Peru and Venezuela); Table S1: Summary of the studies included.
