# Supplementary figures and images for: Prevalence of Human Intestinal Entamoeba spp. in the Americas: A Systematic Review and Meta-Analysis, 1990–2022
Source: Pathogens. 2022 Nov 16;11(11):1365. doi: 10.3390/pathogens11111365 (PMC9699389; doi:10.3390/pathogens11111365)

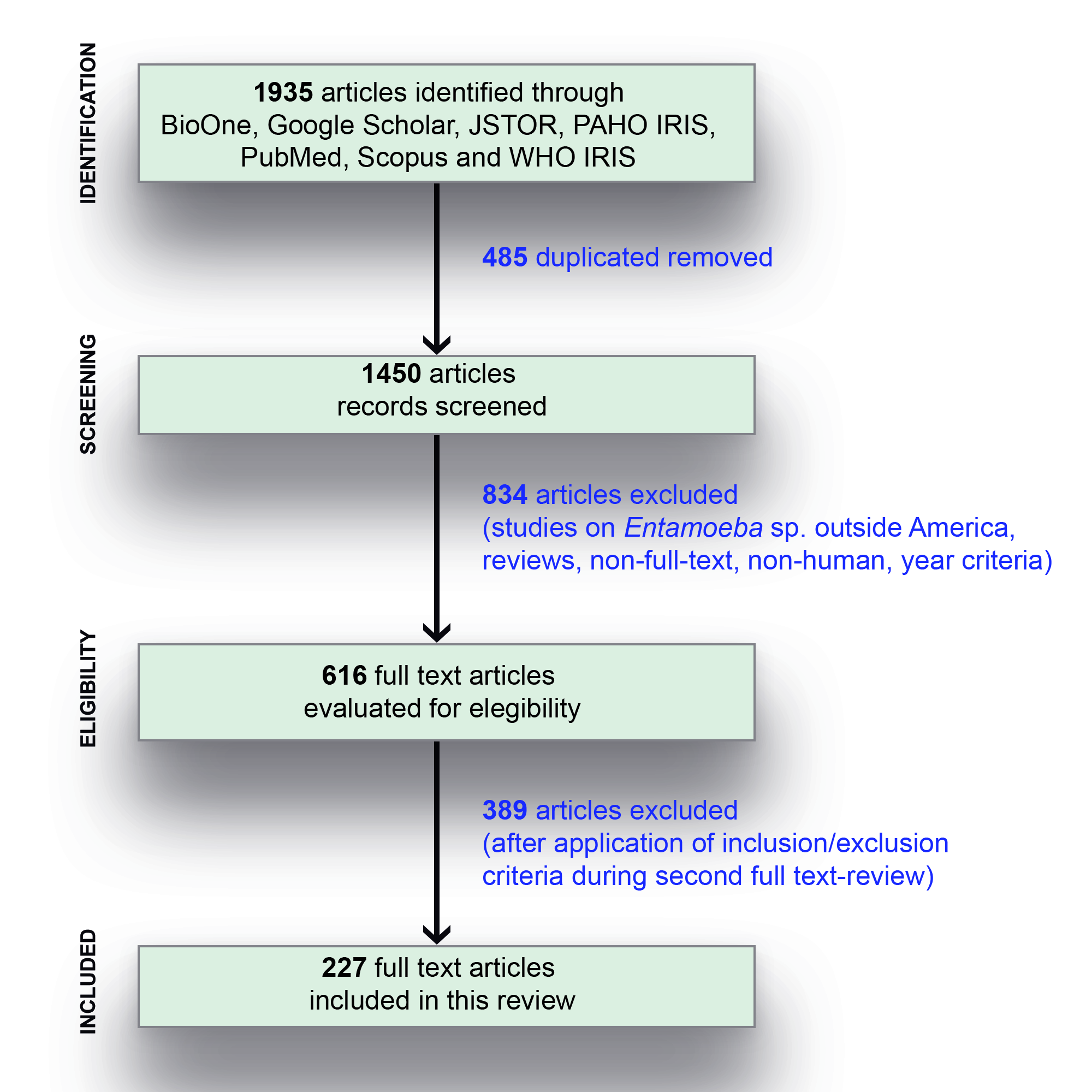

Supplement: Supplementary file 1 [file pathogens-11-01365-s001.zip › Supplementary Figure S1.tif]
